# Lymphocyte subset abnormalities in early diffuse cutaneous systemic sclerosis

**DOI:** 10.1186/s13075-020-02383-w

**Published:** 2021-01-06

**Authors:** David A. Fox, Steven K. Lundy, Michael L. Whitfield, Veronica Berrocal, Phillip Campbell, Stephanie Rasmussen, Ray Ohara, Alexander Stinson, Mikel Gurrea-Rubio, Evan Wiewiora, Catherine Spino, Erica Bush, Daniel Furst, Shiv Pillai, Dinesh Khanna

**Affiliations:** 1 Division of Rheumatology, Department of Internal Medicine, Scleroderma Program, Clinical Autoimmunity Center of Excellence, University of Michigan, Ann Arbor, MI USA; 2 Department of Biostatistics, University of Michigan, Ann Arbor, USA; 3 Department of Biomedical Data Science, Geisel School of Medicine at Dartmouth, Hanover, USA; 4 Division of Rheumatology, UCLA, Los Angeles, USA; 5 Ragon Institute of MIT, MGH and Harvard, 400 Technology Square, Cambridge, MA 02139 USA

**Keywords:** Systemic sclerosis, Autoimmunity, CD4 T cells, Interleukin, CD319

## Abstract

**Background:**

Abnormalities in lymphocyte surface markers and functions have been described in systemic sclerosis (SSc), but conflicting results abound, and these studies often examined patients with heterogeneous disease duration, severity, clinical phenotype, and concurrent immunosuppressive agents. We studied a clinically homogeneous group of early diffuse cutaneous SSc patients not exposed to immunosuppressive drugs who were enrolled in a clinical trial and compared their immune parameters to healthy control subjects.

**Methods:**

Lymphocyte subsets were enumerated by multi-parameter flow cytometry of peripheral blood mononuclear cells at baseline visit. Production of the cytokines IL-4 and IL-17 was measured by intracellular flow cytometry following T cell activation.

**Results:**

SSc patients had increased percentages of CD4+ T cells but lower percentages of CD8+ T cells versus controls. The CD28-negative population was expanded in SSc, in the CD4 subset. Striking expansion of CD319+ T cells was noted among the CD4+ cells, in which they were barely detectable in healthy subjects. Frequencies of IL-4 producing cells did not differ between SSc and controls, but expansion of IL-17 producing cells was observed in SSc. A higher proportion of CD319+ cells produced cytokines, compared to other CD4+ cells. Numbers of activated T cells, regulatory T cells, and B cells were similar in SSc and control groups. Circulating follicular helper but not peripheral helper T cells were slightly expanded in SSc.

**Conclusion:**

In this carefully selected group of early diffuse cutaneous SSc patients, analysis of immune cell parameters has identified abnormalities that likely reflect disease pathogenesis and that are candidate biomarkers for sub-classification and targeted treatment. The CD4+CD319+ (SLAM-F7+) cells are cytotoxic and oligoclonal, were recently shown to be a dominant T cell population in perivascular lymphocytic infiltrates in SSc skin, actively secrete cytokines, and are emerging as a target for novel treatments of SSc.

## Background

Systemic sclerosis (SSc) is a complex multisystem disease characterized by autoimmunity, vascular dysfunction/damage, and fibrosis of target organs that may include the skin, lung, heart, kidney, and gastrointestinal tract [1]. CD4+ T cells are central to the pathogenesis of a range of autoimmune diseases, both through their role in activating B cell differentiation and autoantibody production and through secretion of cytokines. Substantial evidence supports the concept that T cells play a key role in the pathogenesis of early SSc, including cutaneous disease, and at least some of the visceral complications. Skin biopsies obtained from SSc patients early in their disease demonstrate a perivascular, mononuclear cell infiltrate comprised of T cells and macrophages [2]. T cells are the dominant population of lymphocytes in the skin and are activated [3]. T cell infiltration correlates with skin involvement, suggesting a relation between inflammation and fibrosis [4]. Recent data using nextGen RNA Sequencing in 57 subjects with early diffuse cutaneous SSc (dcSSc, mean disease duration of 1.3 years) shows that the adaptive immune system signature was present in a majority of the subjects: CD8+ T cell, CD4+ T cell, and B cell signatures were present in 67%, 61%, and 67% of patients, higher than previously published data in long-standing disease [5].

Many issues remain unresolved about the roles of various lymphocyte subsets in the pathogenesis of SSc, including the importance of Th17 and Th2 effector cell subsets of CD4+ T cells, defects in regulatory T cell numbers and function, and whether other T or B cell subsets differ meaningfully from their numbers and function in healthy controls. These uncertainties may in part arise from a study of patients with heterogeneous duration of disease, different forms of SSc, and prior or ongoing exposure to cytotoxic immunosuppressive drugs and biologic agents.

We sought to define the lymphocyte profile of early dcSSc in patients whose immune parameters were not confounded by a variable degree of internal organ involvement, long disease duration, or effects of medications. We therefore studied subjects enrolled in a phase 2 trial of abatacept in early dcSSc, comparing their baseline lymphocyte subsets and effector T cell function to healthy control lymphocytes that were analyzed concurrently. Multiple abnormalities were observed in dcSSc, notably an expanded population of CD4+CD319+ lymphocytes that may play an important role in the early pathogenesis of SSc.

## Methods

### Study participants

Patients with dcSSc were participants in the ASSET study, which was a randomized placebo-controlled double-blind trial of abatacept [6]. Blood samples were collected at baseline, 1 month, 3 months, and 6 months during the course of the ASSET study. The data reported herein encompass baseline analyses of lymphocyte subsets from all 88 patients enrolled in this trial.

Clinical results of the ASSET study have been reported [6]. Briefly, key inclusion criteria were [1] adult participant, age 18 and older [2]; diagnosis of SSc, as defined using the 2013 American College of Rheumatology/European Union League Against Rheumatism classification of SSc [7], and dcSSc, as defined by LeRoy and Medsger [8]; and [3] disease duration of ≤ 36 months (defined as time from the first non-Raynaud phenomenon manifestation). Oral corticosteroids (≤ 10 mg/day of prednisone or equivalent) and NSAIDs were permitted if the patient was on a stable dose regimen for ≥ 2 weeks prior to and including the baseline visit, but no background immunomodulatory therapies were allowed. Written informed consent was obtained from each participant. All study procedures were approved by institutional IRBs.

A group of 25 healthy controls was studied concurrently, specifically for the analyses contained in this report. These study participants were sex-matched and similar in age to the SSc patients (Tables 1 and 2).

**Table 1:** Demographics of the SSc patients and control subjects

	Control	SSc	*p* value
Female	20	66	> 0.05
Male	6	22	> 0.05
Mean age (range)	42 (22–70)	49 (18–73)	< 0.01

**Table 2:** Demographic and baseline disease characteristics of the SSc patients

	Overall (***N*** = 88)
Age, years, mean (SD)	49 (13)
Female, *N* (%)	66 (75%)
White, *N* (%)	72 (82%)
Not Hispanic or Latino, *N* (%)	76 (86%)
Disease duration, years*, mean (SD)	1.59 (0.81)
Disease < 18 months, *N* (%)	53 (60%)
mRSS, mean (SD)	22.45 (7.65)
FVC% predicted, mean (SD)	85.4 (15.10)
DLCO% predicted, corrected for Hgb, mean (SD)	78.0 (18.24)
Patient global assessment, mean (SD) [theoretical range 0–10]	4.09 (2.38)
HAQ-DI, mean (SD) [theoretical range 0–3]	1.05 (0.71)
Physician global assessment, mean (SD) [theoretical range 0–10]	4.77 (1.67)
Tendon friction rubs, *N* (%)	32 (36%)
Large joint contractures, *N* (%)	63 (72%)
Swollen joint count, mean (SD) [theoretical range 0–28]	3.75 (5.70)
Proportion of participants with ≥ 1 swollen joint count, *N* (%)	42 (48%)
Previous use of immunosuppressives, *N* (%)	12 (14%)
Use of prednisone, *N* (%)	12 (14%)

*mRSS* modified Rodnan skin score, *FVC* forced vital capacity, *DLCO* diffusion capacity of carbon monoxide

*Disease onset was defined as the first non-Raynaud’s sign or symptoms

### Collection and processing of samples

Peripheral blood was collected in heparinized tubes at participating clinical sites and shipped in insulated containers for overnight delivery to the University of Michigan. Peripheral blood collected at the University of Michigan was left overnight at room temperature prior to processing. PBMCs were isolated from heparinized peripheral blood using Histopaque-1077 (Sigma-Aldrich) density gradient centrifugation and washed three times in PBS containing 2% FBS. Aliquots of PBMCs were cryopreserved in FBS containing 10% DMSO and stored in liquid nitrogen until use.

### Cell culture

Cryopreserved PBMCs were thawed in a water bath and suspended in RPMI-1640 containing 10% FBS. Cells for the Th2/Th17 analysis panel (Supplementary Table 1) were plated into 12-well cell culture plates (Corning) pre-coated with anti-CD3 antibody (OKT3) at 1 × 10^6^ cells per well. Anti-CD28 (CD28.2, 5 μg/ml) was added to the wells and the cells were incubated for 24 h at 37 °C. Brefeldin A (5 μg/ml) was added at 20 h. The remaining PBMCs not plated for the Th2/Th17 panel were used immediately for flow cytometry staining.

### Preparation and staining of cells for flow cytometry

PBMCs were washed in flow buffer (PBS containing 0.2% BSA and 5 mM EDTA), passed through a cell strainer (40-μm pore, Corning) to remove aggregates, and plated at 5 × 10^5^ cells per well in 96-well conical bottom plates (Nunc) on ice. Cells were treated with human FcR blocking reagent (Miltenyi Biotec) and DAPI (4′,6-Diamidino-2-Phenylindole, Dihydrochloride, Invitrogen) or Zombie Violet™ (Biolegend) for 15 min on ice and then washed three times in flow buffer prior to antibody labeling. Cell surface molecules were stained with antibodies (Supplementary Table 1) for 20 min on ice, washed twice in flow buffer, and resuspended in 2% formalin (0.2 ml). Cells for intracellular (IC) staining were treated with IC fixation and permeabilization buffer (eBioscience) prior to staining. Cells were stained with IC antibodies (Supplementary Table 1) in IC fixation and permeabilization buffer for 20 min at room temperature. Cells were washed in flow buffer then transferred to 96-well Micronics plates for analysis.

### Data analysis

Flow cytometry was conducted using an LSR I Fortessa flow cytometer (BD Biosciences) with FACSDiva software. Five thousand single color bead controls were recorded and compensation values for each parameter were set. Flow data were analyzed with FlowJo (v10).

Lymphocytes were selected from the center population of PBMCs in the Forward Scatter-Area (FSC-A) vs Side Scatter-Area (SSC-A) plane. From the lymphocyte population, single cells were identified in the FSC-A vs. Forward Scatter-Height (FSC-H) plane. From the unstained control, a gate was set around all cells in the 450/50 (405)-Area vs SSC-A plane to determine live cells and applied to all other samples. For analyses of T cell populations, cells were then gated on CD3 using the CD3 Fluorescence minus one (FMO) to determine gating. For analyses of B lymphocytes, the live singlet lymphocytes were gated as CD19+ cells. Additional gating for subsets within the T and B cell populations were determined based on FMO staining for each of the markers.

Statistical comparisons of patient versus control groups were performed using the Student *t* test. For non-normally distributed data, the Wilcoxon rank-sum test was also used.

## Results

The mean age for the 88 SSc patients was 49 (range 18–73) years, 66 (75%) were female, and mean disease duration (from 1st non-Raynaud’s phenomenon) was 1.59 years. The healthy controls were 77% female and were slightly younger than the SSc patients (mean age 42, range 22–70).

**Fig. 1:**
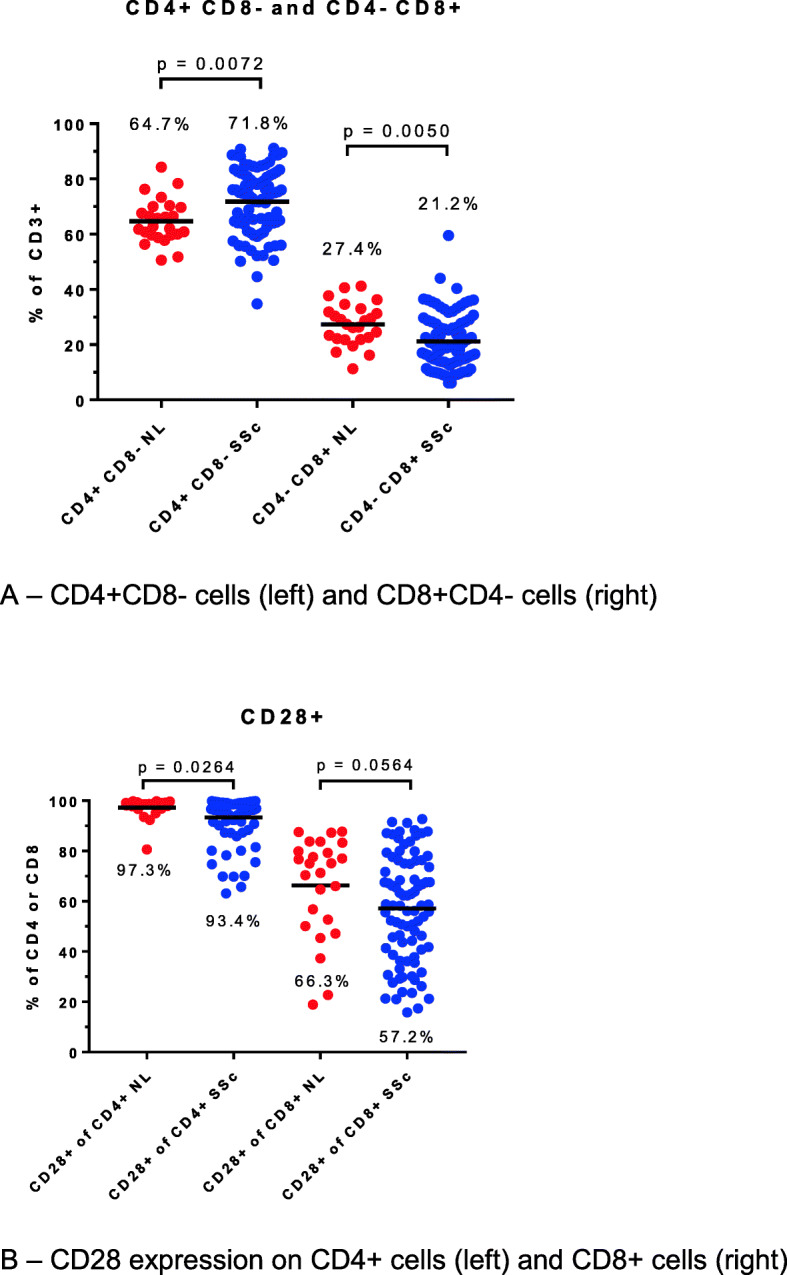
T cell subsets in systemic sclerosis patients and healthy controls. **a** CD4+CD8− cells (left) and CD8+CD4− cells (right). **b** CD28 expression on CD4+ cells (left) and CD8+ cells (right)

Flow cytometry of peripheral blood mononuclear cells, gated on lymphocytes, showed a higher percentage of CD4+ cells in SSc compared to controls. Conversely, the percentage of CD8+ cells was lower in SSc compared to controls (Fig. 1a). SSc patients had a higher percentage of CD28−CD4+ cells compared to controls (Fig. 1b; all *p* < 0.05).

**Fig. 2:**
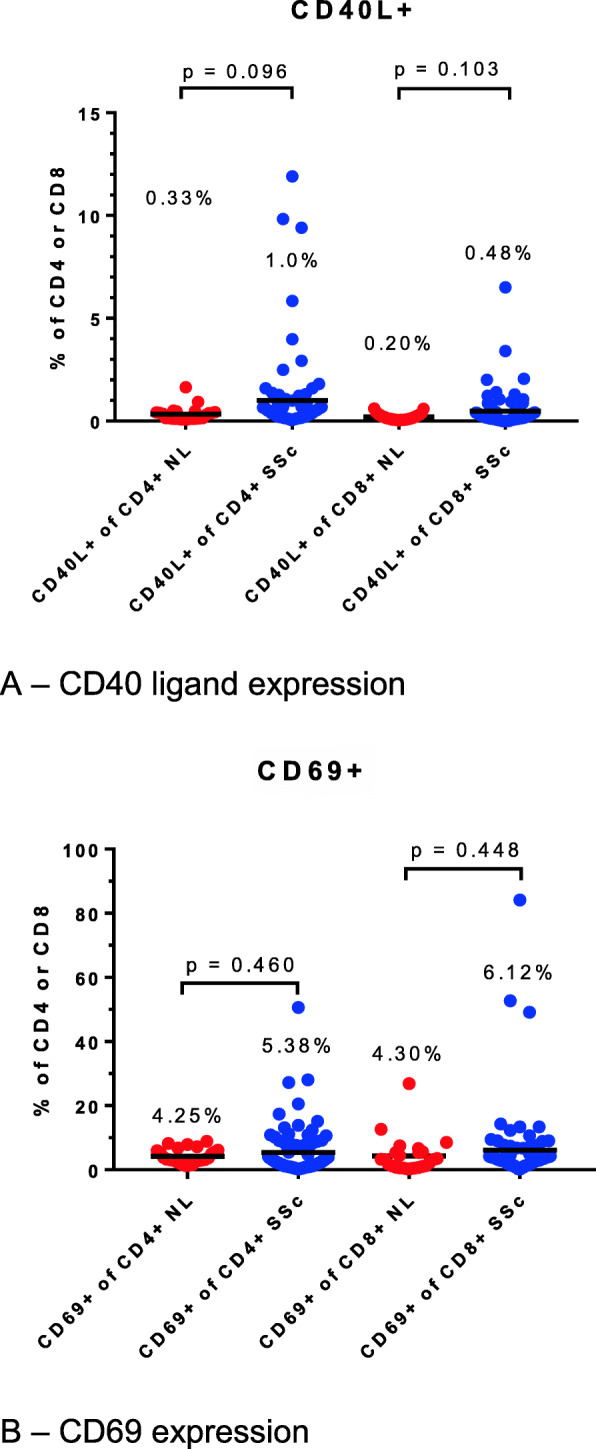
Expression of T cell activation markers. **a** CD40 ligand expression. **b** CD69 expression

Expression of CD40 ligand (CD40L) and CD69 is upregulated upon T cell activation. A few patients with SSc had elevated expression of these molecules on circulating T cells above the range seen in the healthy controls, but the SSc group overall did not show significantly elevated expression of either CD40L or CD69 (Fig. 2).

**Fig. 3:**
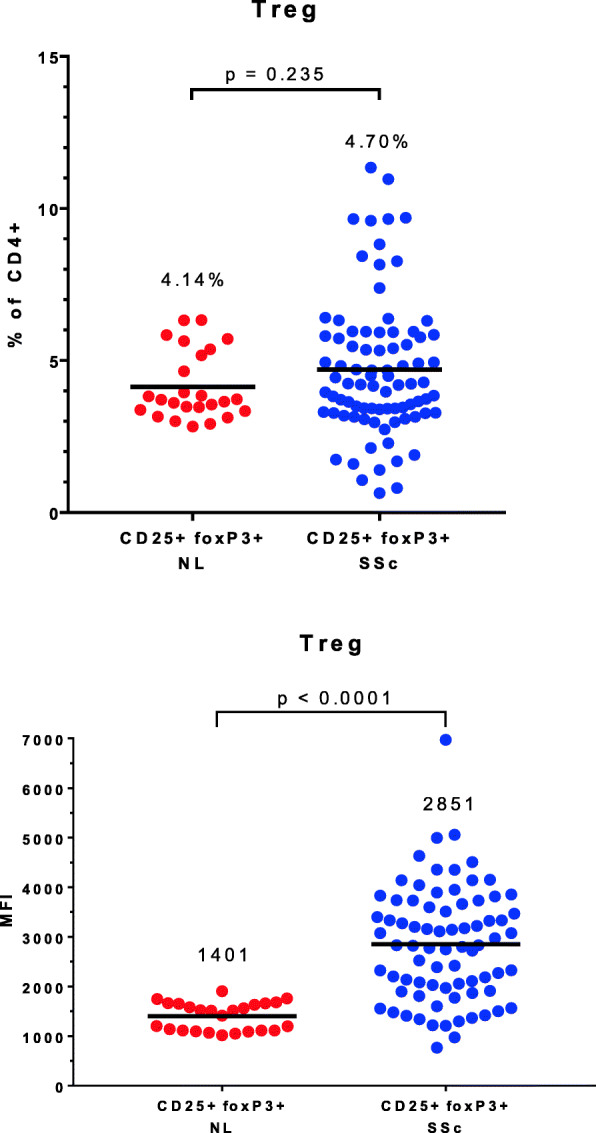
Regulatory T cell numbers in systemic sclerosis compared to healthy controls

Regulatory T cells (Treg) are defined by co-expression of CD25 (a subunit of the interleukin 2 receptor) on the cell surface and intracellular expression of FoxP3, the canonical Treg transcription factor. Deficiencies in the numbers and/or function of Treg have been described in SSc [9, 10] and in other systemic rheumatic diseases [11]. However, in our clinically homogeneous population of patients with early dcSSc, the Treg number did not differ between SSc and control samples, although the range was greater in the SSc group (Fig. 3). Furthermore, the intensity of expression of FoxP3 was significantly greater in the SSc patients than in the control group (mean fluorescence intensity 2851 versus 1401, *p* < 0.0001). We also measured the frequency of resting and activated Tregs in a subset of the subjects (6 patients and 6 controls) and observed no differences between the groups. As a percentage of CD4+ cells, the results were resting T regs (CD45RA+FoxP3-low) in 4.25% in controls, 3.63% in patients, *p* = 0.24; and activated Tregs (CD45RA−FoxP3-high) in 2.61% in controls, 2.62% in patients, *p* = 0.97.

**Fig. 4:**
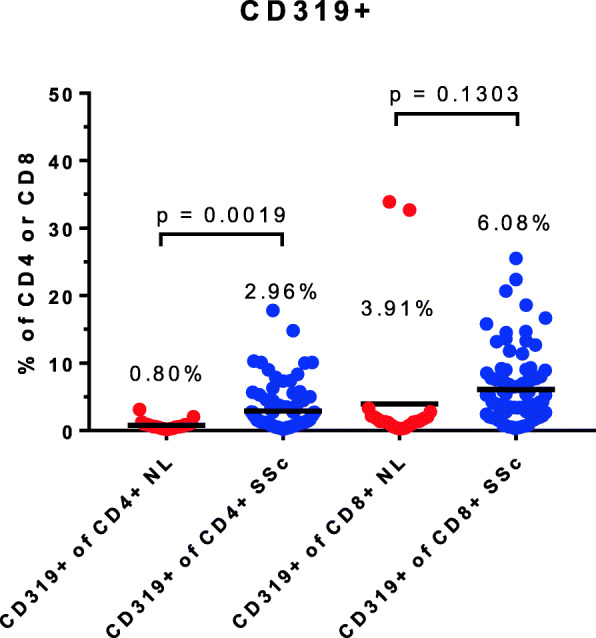
Expansion of the cytotoxic CD4+CD319+ cell population in systemic sclerosis

The surface marker CD319, also known as SLAM F7, is normally expressed by natural killer cells, a subset of CD8+ lymphocytes and plasma cells, but by only a tiny number of normal CD4+ cells. Recently, an expansion of this subset has been noted in IgG4 disease which, like SSc, has a prominent component of fibrosis [12–14]. On non-B lineage cells, CD319 is a marker for lymphocytes with cytotoxic capabilities. We observed the presence of a small subset of CD4+CD319+ cells in SSc (mean 2.96%), which was considerably expanded compared to the control group, and this difference was highly statistically significant (Fig. 4). Among CD4+ cells, most SSc patients had a CD319+ population more numerous than the upper boundary of the range seen in healthy controls.

Based on the pattern of gene expression in skin biopsies, patients with SSc can be subdivided into three molecular subsets, termed inflammatory, fibroproliferative, and normal-like. Patients enrolled in the ASSET study included individuals in each of these categories, based upon analysis of skin biopsies performed at entrance into the study [6], The percentage of CD319+ cells among the CD4+ T cells was not significantly different in the 3 groups (inflammatory 3.5%, fibroproliferative 2.9%, normal-like 2.4%; *p* > 0.05 between any 2 of these groups). It is possible that the inflammatory subset has a greater number of CD319+CD4+ cells in total throughout the body, since migration of large numbers of these cells into the skin did not deplete the percentage in blood. Similarly, the percentage of CD4+ cells that were CD28-negative was not different between the groups (inflammatory 7.76%, fibroproliferative 7.46%, normal-like 6.03%).

**Fig. 5:**
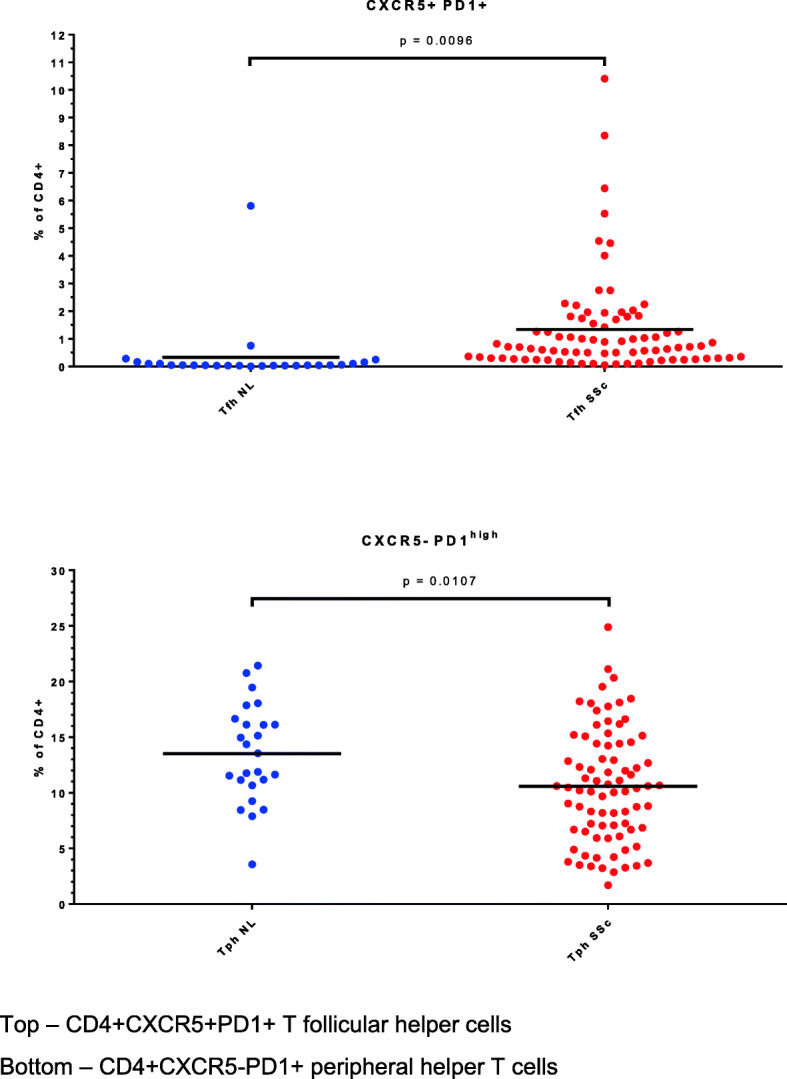
T helper cell populations in systemic sclerosis and healthy control peripheral blood. Top—CD4+CXCR5+PD1+ T follicular helper cells. Bottom—CD4+CXCR5−PD1+ peripheral helper T cells

SSc is characterized by the presence of autoantibodies, as well as by T cell rich lymphocytic infiltrates in target organs such as the skin. Circulating T cell populations of importance in the generation of antibody responses include T follicular helper (Tfh) cells and the recently described T peripheral helper (Tph) cells [15]. Tfh cells are CD4+CXCR5+PD1+ while Tph cells are CD4+CXCR5−PD1^high^. Each of these subsets was detectable in SSc peripheral blood (Fig. 5), with more Tfh but fewer Tph in SSc patients compared to controls. We also measured expression of the chemokine receptors CCR2 and CCR7 on Tph and Tfh cells. In Tph cells, we found no differences between patients and controls. However, in circulating Tfh cells, the %+ for CCR2 was significantly lower in the patients (59%, compared to 77% in the controls—supplementary figure). Nearly all Tfh cells expressed CCR7, mean > 98%+ in both patients and controls.

**Fig. 6:**
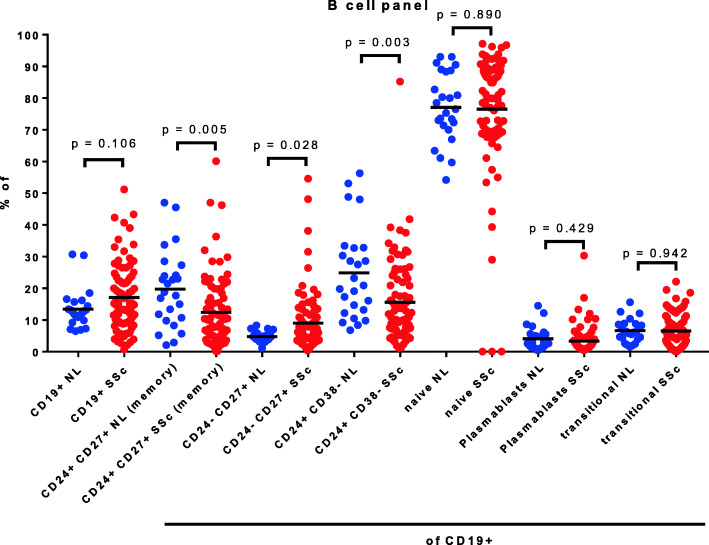
B cell subsets in systemic sclerosis and control peripheral blood

The mean percentage of B lymphocytes (CD19+) in SSc samples was not significantly greater than in the controls, but more striking heterogeneity was observed in the SSc group (Fig. 6). B cells, like T cells, can be further divided into functional subsets by expression of surface structures. In this study, the CD19+ populations were subdivided using CD27 expression into naïve B cells (CD27neg) and memory (CD27+) B cells. Within the naïve subset, an immature transitional (CD24highCD38high) population that has been reported to have immune regulatory function was measured. Memory B cells were further classified as CD24 positive and negative memory subsets, and the plasmablast subset (CD27+CD24lowCD38+) was also measured. The largest differences in B cell subsets between controls and SSc patients were found within the CD24+ memory population and in a CD24+CD38neg subset, both of which were significantly lower in SSc patients (Fig. 6).

**Fig. 7:**
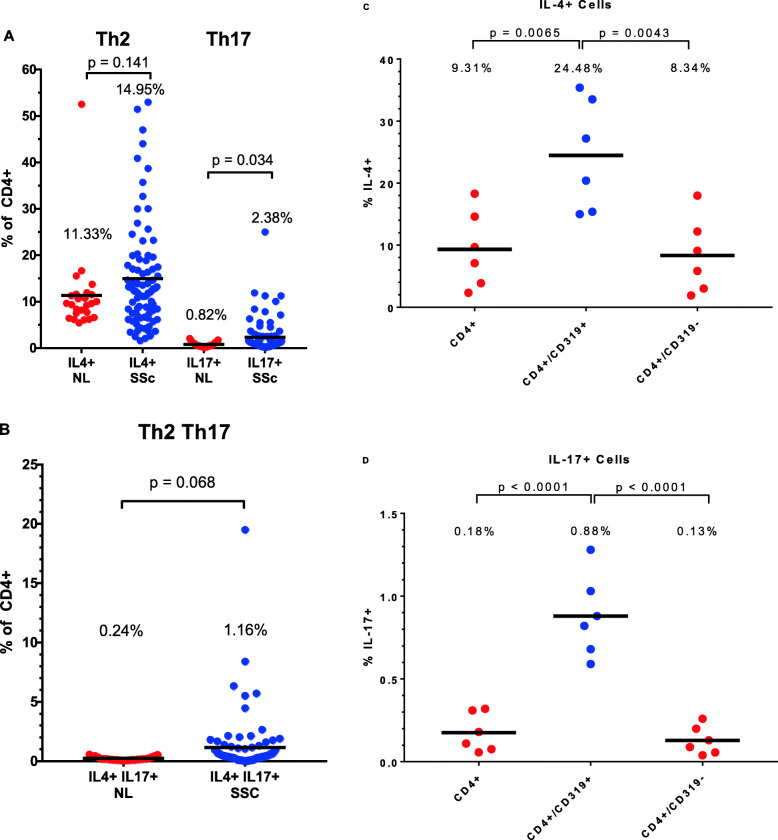
Th2 and Th17 cytokine production following T cell activation. The *p* values shown were calculated using the Student *t* test. Using the Wilcoxon rank-sum test, the *p* values were 0.071 for Th2 cells, 0.007 for Th17 cells, and 0.023 for Th2Th17 cells, when comparing the SSc and control groups

The data presented thus far describe lymphocyte populations identified by various subset markers, but do not directly measure functions of these cells. To measure the capacity of circulating T cells to produce cytokines viewed as relevant to the pathogenesis of SSc, PBMCs were activated in 24-h cultures using stimuli directed at T lymphocytes, and the synthesis of IL-4 and IL-17 was detected by intracellular flow cytometry gated on CD4+ cells. More T cells could be classified as Th2 (IL-4 producers) than Th17 (IL-17 producers) (Fig. 7a). SSc patients did not differ from healthy controls regarding the numbers of Th2 cells, but did show expansion of Th17 cells. A small population of Th2Th17 double-lineage cells that produced both IL-4 and Il-17 was more prominent in a subset of the SSc patients. The difference between the control and SSc groups was not statistically significant by the *t* test but was by the Wilcoxon rank-sum test (Fig. 7). We also examined cytokine production by CD319+ T cells in a subgroup of 6 patients. The frequency of cytokine production (IL-4, IL-17, and also interferon-gamma) was significantly higher by CD319+CD4+ cells compared to total CD4+ cells (Fig. 7), but not by CD319+CD8+ cells compared to total CD8+ cells.

## Discussion

Our study has several strengths and some limitations. An important feature of this study was the enrollment of a SSc cohort that was more clinically homogeneous than most prior groups of SSc patients whose immune parameters were investigated. Specifically, all of our patients had early and active dcSSc (mean disease duration, 1.59 years) without clinically significant organ involvement. Moreover, none of these patients had been treated with cytotoxic or biologic medications, and the use of low-dose corticosteroids was minimal (14% at baseline). The absence of confounding factors due to medications or clinically significant organ involvement may account for some of the differences between our results and those from prior reports. Efforts to control for these confounding factors while analyzing lymphocyte subsets in patients with longstanding SSc who were treated with cytotoxic medications were only partly successful [16]. The analysis of patients with early disease may provide better clues to abnormalities in the immune and inflammatory pathways that lead to tissue damage and fibrosis in SSc, versus phenomena that are the consequence of fibrosis. The control samples were sex-matched. Although the control subjects were slightly younger than the SSc patients, the magnitude of the age difference was modest. Moreover, comparison of the younger versus older subjects in the control group did not identify age-associated changes that would confound the comparison with the SSc group (data not shown).

The patient samples were processed and handled in a uniform fashion, as were the control samples. This involved cryopreservation of all samples, which allowed batch thawing, processing, and analysis with multiple internal controls and standards to achieve uniformity in the flow cytometry methodology. Cryopreservation may theoretically lead to non-uniform loss of cells from various lymphoid subsets, but we did not observe such phenomena in comparing fresh versus cryopreserved samples from a subset of the control subjects (data not shown). Moreover, the thawed cells were readily activated to produce cytokines.

Limitations include a restricted repertoire of functional studies that was performed, based in part on the number of cells available from patient samples. In assessing the integrity of the Treg population, for example, conclusions are ideally based not solely on the enumeration of these cells, but also on measurement of their capacity to enforce suppression of both effector cell proliferation and cytokine production.

We observed several differences between the SSc patients and the controls, some of them striking. The balance of CD4+ versus CD8+ T cells was more skewed towards the CD4+ subset in SSc. A decrease in CD8+ T cells has been previously reported in SSc, in a patient cohort weighted towards long-standing disease [17]. Expansion of the CD28-negative subset was seen in our SSc patients among CD4+ T cells. CD4+ cells that lack CD28 are rare in blood samples from healthy subjects but are more abundant in patients with RA and have been described as oligoclonal, senescent, autoreactive, and pathogenic [18].

T cell activation markers (CD40L, CD69) were over-expressed on only a small number of SSc blood samples, but this data should not be interpreted as contradicting a critical role for T cells in this disease. Expression of these structures on the cell surface can be transient, and activated cells may preferentially migrate into target organs such as skin or traffic back to lymphoid tissues. Thus, the observation of a small subset of activated or functionally unusual T cells in the circulation can correspond to a major role for such cells in sites of disease. A relevant and striking example is the appearance in our group of patients with early dcSSc of a small but significantly expanded subset of CD4+CD319+ lymphocytes, also referred to as SLAMF7+ or cytotoxic CD4+ cells (abbreviated CD4CTL). Moreover, we show here that this CD4+CD319+ subset is significantly enriched for cells that can produce cytokines in short-term cultures. These cells have recently been identified in both IgG4 disease [12–14] and SSc skin tissue in early diffuse and limited cutaneous SSc. In SSc skin, these cells comprise 20–60% of the CD4+ cells and encircle the skin microvasculature, associated with evidence of damage to adjacent endothelial cells. They express and appear to locally discharge contents of cytotoxic granules (perforin and granzymes) [19], explaining the cellular origin of these tissue-damaging mediators that were first noted in SSc skin lesions decades ago [20].

Tregs control immune responses through multiple mechanisms, and defects in Treg numbers and/or functions have been described in rheumatoid arthritis (RA), systemic lupus erythematosus, and SSc [9, 10, 21]. In SSc, increased, decreased, and normal numbers of Tregs have been reported in various studies, but function of circulating Tregs was consistently found to be deficient. In some situations, Treg defects can be reversed by successful treatment of autoimmune disease, particularly RA, by neutralization of cytokines (such as TNF or IL-6) that can impair Treg differentiation and/or functional effectiveness [22], but similar studies have not been reported in SSc. We found no difference in the mean numbers of total, activated, or resting Tregs between SSc and control subjects, although the range was much greater in the SSc samples, and many SSc patients had numbers of Tregs greater than or less than the limits of the range observed in the controls. The demonstration of normal Treg numbers does not establish that their function is intact and we did not assess this in the current analysis. Nevertheless, the unexpected finding of increased intensity of FoxP3 expression in Tregs of the patients argues against a functional Treg defect in patients with early, untreated SSc. Moreover, it is possible that Treg cytokines could contribute to a fibrotic diathesis in SSc.

In SSc, pathogenic roles have been attributed to both Th17 and Th2 cells, which secrete IL17 and IL4, respectively [21, 23–26]. The number of cytokine-producing Th17 but not Th2 cells was modestly higher in this study in cultures of mononuclear cells from SSc versus controls. In addition, a small population of double-producers of IL4 and IL17 was expanded in some SSc patients. The existence of such cells is somewhat paradoxical, as IL4 typically suppresses Th17 development and cytokine secretion. However, such cells have been described in asthma [27]. In mice, antigen presentation in the lung can lead to the appearance of such cells, which originate from Th17 and not Th2 precursors [28]. Whether their presence in early diffuse SSc is a biomarker for subsequent pulmonary deterioration will require longitudinal studies. One report suggests that SSc patients with lung fibrosis had higher IL4 production by peripheral blood lymphocytes [29].

Autoantibody production in autoimmune diseases such as SSc generally requires T helper cell collaboration with autoantigen-specific B lymphocytes. Tfh cells in germinal centers of lymphoid organs are very important for B cell activation, differentiation, and antibody production and are present in the circulation in small numbers. We observed expansion of this subset in our SSc patients, as did a recent study that indicated Tfh expansion in a SSc cohort with average disease duration of 12 years [30]. In circulating Tfh cells the %+ for CCR2 was significantly lower in the patients (59%), compared to 77% in the controls. CCL2, a ligand for CCR2, is elevated in early systemic sclerosis and is a biomarker for progression of interstitial lung disease in these patients [31]. The lower percentage of CCR2+Tfh in the patients could indicate CCL2-directed migration of such cells into lymphoid tissues and/or target organs in systemic sclerosis.

Recently, a related T cell subset with a slightly different surface marker profile (lacking CXCR5 but expressing PD-1) has been described [15]. This subset, termed Tph (T peripheral helper) cells, are abundant in RA, both in the target organ (synovial tissue) and in the blood [32]. Expansion of this subset was also recently reported in SLE peripheral blood [32]. In each of these studies, the majority of patients had long-standing disease. Increased expression of PD-1 was found in a heterogeneous cohort of SSc patients with an average disease duration of 7.3 years (range 1 month–42 years) [33]. We detected fewer Tph cells in SSc blood than in the controls, although the numerical difference was modest. Longitudinal studies and functional analyses will be required to assess the importance of these observations.

Differences between SSc and control samples were also found using a panel of antibodies to B cell surface markers. The range of the percent of B cells among total lymphocytes was far more heterogeneous than in the controls. Among the various B cell subsets, there was a significant decrease in the percentage of CD24+CD27+ memory B cells in SSc patients compared to the controls. There was also an increase in the CD24−CD27+ B cell subset, which contains activated memory B cells.

Longitudinal analysis of lymphocyte surface markers and function would potentially yield further insights and were performed on blood samples from the SSc patients but not the normal controls. These longitudinal results will be reported separately, comparing abatacept versus placebo-treated groups in the ASSET study. Further understanding of SSc, and especially of how to move towards a precision medicine approach that matches molecular subsets with ideal therapeutic targets, will hopefully emerge from detailed analysis and correlation of multiple immune, molecular, and clinical parameters in both cross-sectional and longitudinal studies.

## Conclusion

A clinically homogeneous population of patients with early dcSSc was studied, whose immune parameters were not confounded by long disease duration, exposure to immunosuppressive drugs, or pre-existing organ damage. A parallel cohort of healthy controls was also studied. Numerous differences were found between the SSc patients and healthy controls in lymphocyte subset numbers and cytokine production. Most striking was expansion and heightened cytokine secretion of CD4+CD319+ cells, which are very rare in the peripheral blood of healthy individuals and which have tissue-damaging capabilities. Other abnormalities included expansion of CD4+CD28− cells, T follicular helper cells, and Th17 cells.

Disease-associated abnormalities of lymphocyte surface markers and function are thus readily identifiable in patients with early dcSSc. Some of the aberrantly expressed cell membrane molecules, especially CD319, are candidates for novel therapeutic approaches with potential for more precise targeting of pathogenic lymphocytes.

## Supplementary Information

**Additional file 1: Table S1.** Antibodies used in flow cytometry analyses. **Fig. S1.** Expression of CCR2 on Tfh cells.

## Data Availability

The data in this paper were collected and retained by the authors.

## Abbreviations

SSc: Systemic sclerosis
CD: Cluster designation
SLAM: Signaling lymphocyte activation molecule
DcSSc: Diffuse cutaneous systemic sclerosis
PBMC: Peripheral blood mononuclear cells
DMSO: Dimethylsulfoxide
PBS: Phosphate buffered saline
Th: T helper cell
Treg: Regulatory T cell
FcR: Receptor for immunoglobulin constant region
IC: Intracellular
IL: Interleukin
Tfh: Follicular helper T cell
Tph: Peripheral helper T cell
