# Correction to: Lymphocyte subset abnormalities in early diffuse cutaneous systemic sclerosis

**DOI:** 10.1186/s13075-021-02459-1

**Published:** 2021-03-04

**Authors:** David A. Fox, Steven K. Lundy, Michael L. Whitfield, Veronica Berrocal, Phillip Campbell, Stephanie Rasmussen, Ray Ohara, Alexander Stinson, Mikel Gurrea-Rubio, Evan Wiewiora, Catherine Spino, Erica Bush, Daniel Furst, Shiv Pillai, Dinesh Khanna

**Affiliations:** 1 Division of Rheumatology, Department of Internal Medicine, Scleroderma Program, Clinical Autoimmunity Center of Excellence, University of Michigan, Ann Arbor, MI USA; 2 Department of Biomedical Data Science, Geisel School of Medicine at Dartmouth, Hanover, USA; 3 Department of Biostatistics, University of California, Irvine, CA USA; 4 Department of Biostatistics, University of Michigan, Ann Arbor, USA; 5 Division of Rheumatology, UCLA, Los Angeles, USA; 6 Ragon Institute of MIT, MGH and Harvard, 400 Technology Square, Cambridge, MA 02139 USA

**Correction to: Arthritis Res Ther (2021) 23:10**

**https://doi.org/10.1186/s13075-020-02383-w**

Following publication of the original article [1], the authors reported errors in the affiliation of authors Michael L. Whitfield, Veronica Berrocal, Evan Wiewiora and Catherine Spino, and an additional affiliation was added for author Veronica Berrocal. The correct affiliations are as follows:

Michael L. Whitfield should be affiliated to "Department of Biomedical Data Science, Geisel School of Medicine at Dartmouth, Hanover, USA".

Evan Wiewiora and Catherine Spino should be affiliated to "Department of Biostatistics, University of Michigan, Ann Arbor, USA".

Veronica Berrocal should be affiliated to "Department of Biostatistics, University of California, Irvine, CA, USA" and “Department of Biostatistics, University of Michigan, Ann Arbor, USA

The original article has been corrected.
