# Supplementary material for: Lymphocyte subset abnormalities in early diffuse cutaneous systemic sclerosis
Source: Arthritis Res Ther. 2021 Jan 6;23:10. doi: 10.1186/s13075-020-02383-w (PMC7789011; doi:10.1186/s13075-020-02383-w)
Supplement: Supplementary file 1 — Additional file 1: Table S1. Antibodies used in flow cytometry analyses. Fig. S1. Expression of CCR2 on Tfh cells. [file 13075_2020_2383_MOESM1_ESM.docx]

**Supplementary Materials**

Supplementary Table 1: Antibodies used in flow cytometry analyses

|  | **Antibody Target** | **Clone** | **Fluorochrome** | **Vendor** |
| --- | --- | --- | --- | --- |
| **Activated T Cell** | CD3 | BW264/56 | PerCP | Miltenyi Biotec |
|  | CD4 | M-T466 | APC-Vio770 | Miltenyi Biotec |
|  | CD8 | BW135/80 | PE-Vio770 | Miltenyi Biotec |
|  | CD28 | 15E8 | APC | Miltenyi Biotec |
|  | CD69 | FN50 | PE | Miltenyi Biotec |
|  | CD40L | 5C8 | FITC | Miltenyi Biotec |
|  | CD319 (SlamF7) | 162.1 | PE/Dazzle™ | BioLegend |
| **Tfh** | CD4 | M-T466 | APC-Vio770 | Miltenyi Biotec |
|  | CD185 (CXCR5) | REA103 | APC | Miltenyi Biotec |
|  | CD278 (ICOS-1) | REA192 | FITC | Miltenyi Biotec |
|  | CD279 (PD-1) | PD1.3.1.3 | PE | Miltenyi Biotec |
|  | CD4 | RPA-T4 | APC-H7 | BD Biosciences |
|  | CD185 (CXCR5) | J252D4 | PE | Biolegend |
|  | CD192 (CCR2) | EH12.2H7 | APC | Biolegend |
|  | CD197 (CCR7) | G043H7 | PE Cy7 | Biolegend |
|  | CD278 (ICOS-1) | DX29 | PerCP-Cy™5.5 | BD Biosciences |
|  | CD279 (PD-1) | EH12.2H7 | Alexa Fluor™ 488 | Biolegend |
| **Treg** | CD4 | M-T466 | APC-Vio770 | Miltenyi Biotec |
|  | CD25 | M-A251 | PE | BioLegend |
|  | FoxP3 | PCH101 | APC | ThermoFisher |
|  | CD4 | OKT4 | Alexa Fluor™ 488 | Biolegend |
|  | CD25 | BC96 | APC | Biolegend |
|  | CD45RA | HI100 | APC-H7 | BD Biosciences |
|  | CD45RO | UCHL1 | PerCP-Cy™5.5 | Biolegend |
|  | FoxP3 | 206D | PE | Biolegend |
|  | CD127 (IL-7Rα) | A019D5 | PE Cy7 | Biolegend |
| **Breg** | CD19 | HIB19 | APC Cy7 | BioLegend |
|  | CD24 | ML5 | PerCP | BioLegend |
|  | CD27 | M-T271 | PE Cy7 | BD Biosciences |
|  | CD38 | HIT2 | PE/Dazzle™ | BioLegend |
| **Th2/17** | CD4 | M-T466 | APC-Vio770 | Miltenyi Biotec |
|  | IL4 | 8D4-8 | PE | BioLegend |
|  | IL17A | CZ8-23G1 | APC | Miltenyi Biotec |
|  | CD4 | OKT4 | PerCP-Cy™5.5 | Biolegend |
|  | CD8 | SK1 | APC-H7 | BD Biosciences |
|  | CD319 (SlamF7) | 235614 | PE | R&D Systems |
|  | IFN-g | 4S.B3 | Alexa Fluor™ 488 | Biolegend |
|  | IL-4 | 8D4-8 | APC | Biolegend |
|  | IL17A | eBio64DEC17 | PE Cy7 | eBioscience |

**Supplementary Figure 1:** Expression of CCR2 on Tfh cells
